# Earth before life

**DOI:** 10.1186/1745-6150-9-1

**Published:** 2014-01-09

**Authors:** Caren Marzban, Raju Viswanathan, Ulvi Yurtsever

**Affiliations:** 1 Applied Physics Laboratory, Univ. of Washington, Seattle, WA, 98105-6698, USA; 2 Department of Statistics, Univ. of Washington, Seattle, WA, 98195-4322, USA; 3 Technome, LLC, 8107 Colmar Drive, Clayton, MO, 63105, USA; 4 MathSense Analytics, 1273 Sunny Oaks Circle, Altadena, CA, 91001, USA

**Keywords:** Genome, Evolution, Origin, Regression, Measurement error, Confidence interval, Prediction interval

## Abstract

**Background:**

A recent study argued, based on data on functional genome size of major phyla, that there is evidence life may have originated significantly prior to the formation of the Earth.

**Results:**

Here a more refined regression analysis is performed in which 1) measurement error is systematically taken into account, and 2) interval estimates (e.g., confidence or prediction intervals) are produced. It is shown that such models for which the interval estimate for the time origin of the genome includes the age of the Earth are consistent with observed data.

**Conclusions:**

The appearance of life after the formation of the Earth is consistent with the data set under examination.

**Reviewers:**

This article was reviewed by Yuri Wolf, Peter Gogarten, and Christoph Adami.

## Background

Sharov [1], and more recently, Sharov and Gordon [2] reported an analysis of data on the evolution of genetic complexity during the history of life on Earth. These two works - hereafter denoted SG - use the functional genome size of major phylogenetic lineages, as a measure of genetic complexity, and show that it has an exponential relationship with the estimated dates of the transitions where these lineages first originated. As such, there exists a linear relationship between the logarithm of genome size (*y*) and the transition date (*x*). SG performed regression on a data set on *y* vs. *x*, and proposed that the *x*-intercept of the fit (i.e., where genome size is zero) provides an estimate for the age of life.

The work was criticized on many levels, ranging from the manner in which the data was produced, to the way in which the data was analyzed. A fundamental problem is the paucity of data over the first 2 billion years or so of Earth’s history, resulting in large uncertainties in functional genome size at specific times. For instance, for prokaryotes, the size of the functional genome is guessed from the smallest present-day prokaryote genome. Exactly when this genome size evolved is a matter of conjecture; although an approximate date can be estimated from molecular clock type evolution rates based on more recent organisms (see e.g., the reviews of [1]), rates of increase of functional genome size could have been very different in the distant past. Fitting the data with an extrapolation based on a single, fixed rate of increase could lead to possibly incorrect conclusions. Likewise, the use of only coding regions of the genome as a measure of genome complexity has been pointed out as a potential problem, as non-coding regions could play a regulatory role and the associated complexity is unaccounted for when only coding regions are measured. Thus estimating genome complexity of extinct organisms based on an uncertain estimate of functional genome size of present-day organisms could be doubly flawed.

In addition to all of the above criticisms, there are additional concerns over the statistical analysis in SG. First, and foremost, is the way in which the regression fit is used to extrapolate far beyond the range of *x* values appearing in the data. It is well known that extrapolation can lead to misleading conclusions [3]), and so, any conclusions regarding the age of life, based on extrapolation, should be considered with extreme caution. A second aspect of the SG regression fit is that it does not incorporate statistical uncertainty due to sampling variability, e.g., through confidence or prediction intervals. The inclusion of such intervals can lessen the misleading impacts of extrapolation, because they generally widen as one moves away from the mean of the data [4]. When an interval estimate is produced for the *x*-intercept, then the age of life can be estimated to within a range of possible values. Values outside of the interval may be rejected (with some confidence), but all of the values within the interval are possible, and in fact, equally “likely.” As such, consideration of interval estimates is important because it can mitigate misleading conclusions.

Another limitation of the SG regression analysis is that it does not systematically account for uncertainty in the dates at which the transitions occurred (i.e., the *x*-values of the data), also known as *measurement errors.* As explained here, measurement errors generally reduce the slope of the regression fit, and consequently increase the value of the *x*-intercept. As such, failure to account for measurement errors leads to overestimates for the age of life. Regression models which systematically take measurement errors into account are called *measurement error models*[5,6].

In this paper, a simple measurement error model is developed for the SG data, and rudimentary interval estimates are produced for the fit. In such a framework, the age of life is estimated to be within a range of possible values, with the range itself depending on a quantity proportional to the variance of the measurement errors. In other words, an estimate of the age of life is contingent on an estimate of the typical error in the lineage transition dates. An attempt is made to estimate the variance of the measurement errors, and it is shown that the proposed model is consistent with life having formed around 4.5-billion years ago. In short, we find that when the regression analysis involves interval estimates, and incorporates measurement errors, then the data used by SG provide no evidence to support the claim that life must have formed prior to the formation of the Earth.

## Method

### Regression effect

**Figure 1:**
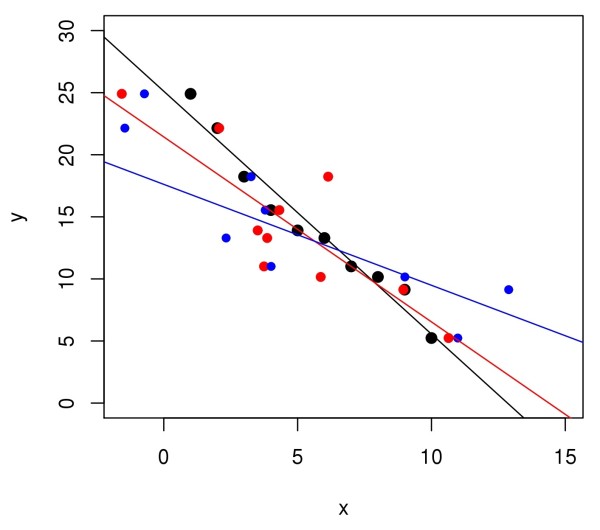
**Demonstration of regression effect.** The black, red, and blue circles show data sets with increasing errors in *x*-values. As a result, their ordinary least-squares fits have progressively smaller slopes.

Consider a scatterplot of *y* vs. *x*, displaying some amount of association between the two variables (e.g., Figure 1). It is well known that as the spread of the data increases, the slope of a least-squares fit approaches zero. This effect is known by a variety of names, including *the regression effect*[7]. It is demonstrated in Figure 1, where the black, red, and blue circles are fictitious data with increasing error in *x*; i.e, the black circles have less scatter than the red circles, etc. The straight lines are the ordinary least-squares fits to the respective data sets. It can be seen that increasing scatter leads to lower values of the slope.

The mathematics underlying the regression effect is straightforward. It is easy to show

(1) $$\frac{ŷ(x)-\bar{y}}{s_{y}}=r(\frac{x-\bar{x}}{s_{x}}),$$

where ŷ(x) is the predicted/fitted value, $\bar{x}$ and $\bar{y}$ are the sample mean of *x* and *y*, respectively, and *s*_*x*_, *s*_*y *_are their sample standard deviations. The quantity *r* is Pearson’s correlation coefficient, and measures the amount of scatter on the scatterplot. As *r* approaches zero (from either side), the predicted value ŷ(x) tends to the sample mean of *y*. Indeed, this “regression to the mean” is the reason why the least-squares fit is called regression [8]. In summary, as the amount of scatter in the scatterplot of *y* vs. *x* increases, the least-squares fit converges to a horizontal line with slope zero, and y-intercept equal to $\bar{y}$.

### Regression dilution

The aforementioned scatter may be due to errors in *x*, in *y*, or both. In the most common form of regression, the predictor *x* is assumed to be error-free, and only the response *y* is assumed to be subject to errors. Measurement error models [5,6] are designed to allow for both *x* and *y* to be subject to errors. Consequently, as expected from the previous example, measurement errors tend to “flatten” the least-squares line - a phenomenon called *regression dilution*[5,6]. Moreover, if the measurement errors can be estimated, then one can undo the dilution.

A simple measurement error model is as follows: Let (*X*_*i*_,*Y*_*i*_),*i *= 1,…,*a*, denote the true, error-free, values of two continuous random variables satisfying the relation

(2) $$Y_{i}=\alpha^{*}+\beta^{*}\;X_{i}\;.$$

The corresponding observed values (*x*_*i*_,*y*_*i*_) can then be written as

(3) $$x_{i}=X_{i}+\omega_{i}\;\;\;,\;\;\;\;y_{i}=Y_{i}+\varepsilon_{i}\;,$$

where *ω*_*i *_and *ε*_*i *_are the measurement error in *X *and the error in *Y*, respectively. For simplicity, it is assumed that both errors are normally distributed with zero mean, and variances given by $\sigma_{w}^{2}$ and $\sigma_{\varepsilon }^{2}$. I.e., $\omega_{i}∼N(0,\sigma_{w}^{2})$ and $\varepsilon_{i}∼N(0,\sigma_{\varepsilon }^{2})$. In the *functional model *the *X *is assumed fixed (non-random), but in the *structural model X *is assumed to be a random variable [5,6]. In other words, in the former, the *a *values of *X*_*i *_are assumed to be fixed quantities, while in the latter they are considered to be a random sample taken from a population (or a distribution). The latter is adopted here because it is more appropriate for the problem at hand, and for simplicity we assume $X_{i}∼N(\mu ,\sigma_{b}^{2})$. The subscripts “b” and “w” are motivated by “between-group” and “within-group” variances - language common to the analysis of variance formulation of regression [9].

If one mistakenly ignores measurement errors (in *X*), and instead performs regression on (*x*_*i*_,*y*_*i*_), i.e.,

(4) $$y_{i}=\alpha +\beta \;x_{i}+\varepsilon_{i}\;,$$

then it can be shown that the least-squares estimate of the regression slope and y-intercept are given by [5,6,10]:

(5) $$\beta =\beta^{*}/\lambda \;\;,\;\;\alpha =\bar{y}-(\beta^{*}/\lambda )\;\bar{x}\;\;,$$

with

(6) $$\lambda =1+\frac{\sigma_{w}^{2}}{\sigma_{b}^{2}}\;.$$

Given that *λ *> 1, it follows that *β *< *β*^∗^, i.e., the slope is “diluted” relative to the slope that would have been obtained if measurement errors were zero. Therefore, as mentioned previously, measurement errors tend to flatten the least-squares fit, and thereby lead to an overestimate of the *x*-intercept. For the SG data, then, measurement errors lead to an overestimate for the age of life.

Equation (5) implies that one can correct this overestimation by simply multiplying the observed regression coefficient *β* by *λ *[5,6,10]. In other words, the quantity (*β λ*) is an estimator of *β*^∗^. Similarly, the least-squares estimate of *α*^∗^ is $\left(\bar{y}-(\beta \;\lambda )\;\bar{x}\right)$. Note that in a measurement error model of the SG data, the estimate for the age of life is given by the corrected *x*-intercept $\bar{x}-\bar{y}/(\beta \;\lambda )$. In order to make any of these corrections, however, one must estimate *λ*.

Frost and Thompson [11] discuss six methods for estimating *λ *and the corresponding variance. One of the simpler methods examined there identifies *λ *as the inverse of the intraclass correlation coefficient (also known as the reliability ratio). One advantage of that estimator is that its variance has a simple expression:

(7) $$\frac{{\left(\lambda^{2}-1\right)}^{2}}{a}\;.$$

In the following not only an attempt is made to estimate *λ *itself, we also consider the “inverse problem” of finding the range of *λ *values which lead to *x*-intercepts consistent with 4.5 billion years as the age of life.

It is not necessary to find a specific *λ* value which leads to an *x*-intercept of 4.5 billion years. A regression fit whose confidence or prediction interval includes an *x*-intercept of 4.5 billion years is sufficient, in the sense that it does not contradict the null hypothesis that life began after the formation of the Earth. In order to construct such an interval, one must compute the variance for the corrected regression slope, a quantity which has been derived in [11]:

(8) $$V[\;(\beta \;\lambda )]=\lambda^{2}V[\;\beta ]+\frac{1}{a}(\beta^{2}+V[\;\beta ]){\left(\lambda^{2}-1\right)}^{2}\;.$$

where Equation (7) has been used.

There is an ambiguity in whether the appropriate interval for this problem is a confidence interval or a prediction interval [4]. The former is designed to cover the true conditional mean of *y*, given *x*, a certain percentage of time, e.g., 95%. The latter is designed to cover a single prediction of *y*, a certain percentage of time. By construction, the prediction interval is wider than the confidence interval. An argument in favor of using a prediction interval is that the *x*-intercept corresponds to a single prediction of *y*. One can also argue that the appropriate interval is a confidence interval, because the *x*-intercept is technically a population parameter. The choice between the two intervals is of secondary importance. What is more important than the choice of the two intervals is that *some* interval must be considered. Here, a prediction interval is used; using confidence intervals leads to qualitatively similar conclusions.

The construction of prediction intervals in measurement error models is itself a complex issue and is considered by [12]. One relatively simple 95% prediction interval is given by $ŷ(x)\pm 1.96\sigma_{\text{pe}}$, where $\sigma_{\text{pe}}^{2}$ is the variance of the prediction error, given by

(9) $$\;\sigma_{\text{pe}}^{2}=\sigma_{\varepsilon }^{2}+\frac{\sigma_{\varepsilon }^{2}}{a}+{\left(X-\bar{X}\right)}^{2}V[\;\beta \lambda ]+[\;{\left(\beta \;\lambda \right)}^{2}+V[\;\beta \;\lambda ]]\;\sigma_{b}^{2}\;\;,$$

where *V *[ (*βλ*)] is given by Equation (8), and $\sigma_{\varepsilon }^{2}$ is estimated by the variance of the residuals. This is the expression derived in [12] for the special case where the value of *X* at which the prediction is made is a known (non-random) quantity. The first three terms on the right-hand side of Equation (9) are the variance of the prediction error in the error-free case [10]; the last term is the result of measurement errors.

### Estimating measurement errors

One may wonder what is a typical value of *λ* for the data at hand. Given Equation (6), *σ*_*b *_and *σ*_*w *_must be estimated. To that end, consider a situation where each *X*_*i *_is measured *n* times. Denoting the resulting data as *x*_*ij*_,*i *= 1,…,*a*, *j *= 1,…,*n*, it is known that unbiased estimates of $\sigma_{b}^{2}$ and $\sigma_{w}^{2}$ are

(10) $$(\frac{s_{b}^{2}}{n}-\frac{s_{w}^{2}}{n})\;\;,\;\;s_{w}^{2}\;,$$

respectively, with $s_{b}^{2}$ and $s_{w}^{2}$ defined as

(11) $$\;s_{b}^{2}=\frac{n}{a-1}\overset{a}{\underset{i}{\sum }}{\left(\bar{x_{\mathrm{i.}}}-\bar{x_{\mathrm{..}}}\right)}^{2}\;\;,\;\;s_{w}^{2}=\frac{1}{a(n-1)}\overset{a,n}{\underset{i,j}{\sum }}{\left(x_{\text{ij}}-\bar{x_{\mathrm{i.}}}\right)}^{2}\;\;.$$

where an overline denotes averaging over the index with a dot [9]. For large *n*, the quantity $s_{b}^{2}/n$ converges to the sample variance of the *X*_*i*_, i.e., $s_{X}^{2}=\frac{1}{a-1}\overset{a}{\underset{i}{\sum }}{\left(X_{i}-\bar{X}\right)}^{2}$, which in turn can be estimated with the sample variance of the *x*_*i*_. For the data at hand $s_{b}^{2}/n∼1.86$ (billion years)^2^. In the large-*n *limit, the term $s_{w}^{2}/n$ converges to zero, because in that limit $s_{w}^{2}$ itself converges to the constant $\sigma_{w}^{2}$. Therefore, asymptotically, $\sigma_{b}∼\sqrt{1.86}∼1.36$ billion years.

The within-group standard deviation *s*_*w *_reflects the spread in values or uncertainty of the dates of appearance of the respective functional genomes (e.g., prokaryote, eukaryote, worms, fish, mammals). While the statistical analysis presented here assumes that the *a *measurements all have common variance (i.e., homoscedastic), in reality the uncertainty in the time of appearance of a functional genome increases from present to past. Thus the largest errors or uncertainties are found in the oldest functional genome considered. As an example of dating uncertainty, while the earliest mammals are believed to have arisen about 225 million years ago, based on early fossils [13], molecular clock studies based on genomes place mammalian origins around 100 million years ago [14]. There is thus an uncertainty of the order of 100 million years or more in setting the time of the mammalian functional genome. The origin of eukaryotes has been identified to lie in the time interval between 2.3 billion and 1.8 billion years ago, thus with an uncertainty of 250 million years around the mean estimate of 2.05 billion years ago [15]. Early fossil evidence for prokaryotes in lava beds has been dated to a time around 3.5 billion years ago [16]; however, it is unclear exactly when the functional genome size reached the present-day minimum value of around 5×10^5^; the uncertainty in this time value could easily be of the order of 1 billion years.

Within-group standard deviations in the dates at which respective functional genome sizes were attained, therefore, have an order of magnitude spread in range of values, from 100 million to 1000 million years, with most standard deviation values being of the order of a few hundred million years. In a homoscedastic model of the type assumed in the present article, we will use as a rough (weighted) estimate a value of *s*_*w *_∼ 500 million years.

Therefore, with *σ*_*b *_∼ 1.36 billion and *σ*_*w *_∼ 0.5 billion, we have *λ *∼ 1.14. For uncertainties around 100 million years, *λ *is around 1.00, and it is around 1.54 if uncertainty is around 1 billion years.

## Results and discussion

The formula for the prediction interval shown above depends on the quantity *λ*. In the previous subsection we arrived at a rough estimate for that quantity. Now, we examine the range of *λ* values which lead to conclusions consistent with the hypothesis that life did not begin prior to the formation of the Earth.

**Figure 2:**
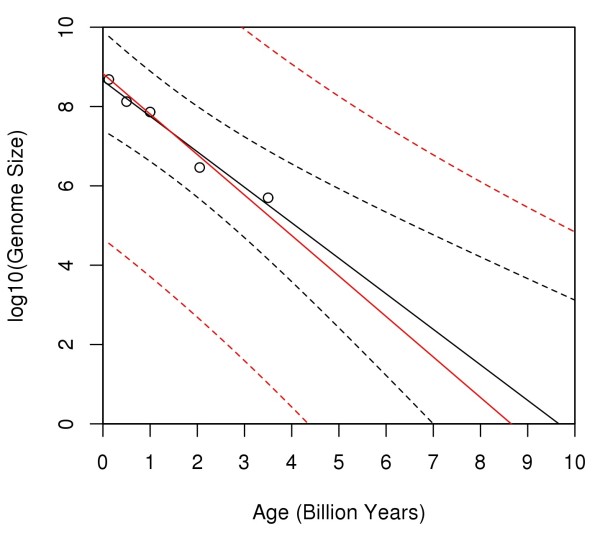
**Regression dilution.** The circles denote the data from SG, and the black, solid line is the ordinary least-squares fit to that data. The *x*-intercept of this line (about 9.5 billion years), is the estimate for the age of life according to SG. The region between the black, dashed curves is the 95% prediction interval for the fit. The red, solid line is the least-squares fit according to a measurement error model with *λ *= 1.14; the red, dashed curves outline the 95% prediction interval/region. Note that this region includes 4.5 billion years (i.e., the Earth’s age).

Figure 2 shows all of the results. The black line shows the ordinary least-squares fit to the data. It is the *x*-intercept of this line, i.e., about 9.5 billion years, which led SG to conclude that life must have begun prior to the formation of the Earth (i.e., about 4.5 billion years ago). The region between the black, dashed curves is the 95% prediction interval for the ordinary least-squares fit. According to this prediction interval (without taking measurement errors into account) life may have originated as early as 7 billion years ago.

The analogous results based on the above measurement error model are shown in red. The value of *λ* for this fit is 1.14 - the value estimated in the previous section. Note that the resulting prediction interval includes 4.5 billion. In other words, if *λ* is about 1.14, then the results of the analysis do not reject the hypothesis that life began after the formation of the Earth. Even a *λ* as small as 1.1 leads to results (not shown here) consistent with 4.5 billion years as the age of life.

Although, a proper interpretation of prediction intervals correctly draws all focus away from the “center” of the interval, it is possible to arrange for the corrected fit itself to have an *x*-intercept of 4.5. The result is not shown here, but the corresponding value of *λ* is about 2.7. Values of *λ* in the 1.1 to 2.7 range are not uncommon; Frost and Thompson [11] even consider *λ* values as large as 5. More importantly, that range includes the *λ* values estimated in the previous section.

Based on Figure 2, note that the genome size at Age = 0 may be as low as 10^5^ (i.e., the lower limit of the prediction interval at Age = 0); one may be inclined to conclude that such a low value is unlikely. Also note that Age = 4.5 is only at the lowest limit of the possible *x*-intercepts; one may then be inclined to conclude that Age = 8.5 (the *x*-intercept of the red line itself) is the more likely estimate for the age of life. Although both premises are correct, the conclusions do not follow. The fallacy in both arguments is to attribute a likelihood to different regions within the prediction intervals. The correct interpretation of prediction (and confidence) intervals does not allow that type of interpretation [4]. Specifically, regarding Figure 2, all one can conclude is that about 95% of prediction intervals will cover genome size, at a given value of Age. More loosely stated, one can be “confident” that the true fit lies *somewhere* within the region between the red, dashed curves; nothing can be said about which of the possible fits (or their slopes and intercepts) are more, or less, likely.

The original conclusion of SG is a consequence of an incomplete analysis. Although the analysis presented here is more complete, many improvements are possible. For instance, nonlinear fits can made, and more refined measurement error models can be developed. The inference component of our analysis (i.e., the 1.96 appearing in the prediction interval formula) can also be improved upon. The assumption of homoscedasticity can be relaxed, the *σ*_*w *_and the *σ*_*b *_can be estimated without the large-*n* assumption, and one can even compute confidence and/or prediction intervals for the *x*-intercept itself. Lastly, data sets considerably larger than used by SG can be employed as genome size data is readily available for many major transitions along the tree of life (see, e.g., http://itol.embl.de/itol.cgi and http://www.ncbi.nlm.nih.gov/genome).

In short, many aspects of the above formulation are simplistic, approximate, or even controversial. As such, they offer avenues of further research. These limitations have not been of concern here because the main goal of the paper has been to introduce measurement error models and to highlight the importance of producing interval estimates of the fit. The details of the measurement error model, the manner in which the interval estimates are generated, or whether the appropriate interval is a confidence or prediction interval, are all of secondary importance because they affect the conclusions only in degree, not in kind.

## Conclusions

A naïve regression model relating genome size to the age of life suggests that life may have formed prior to the Earth’s formation. Here we have shown that measurement errors lead to biased (i.e., over-) estimates for the age of life, and that the bias can be corrected/removed. Additionally, a more refined regression analysis is performed which 1) takes into account measurement errors, and 2) generates interval estimates of the fit. The analysis depends on a parameter, *λ*, which is related to the variance of the measurement errors. We find that a wide range of plausible *λ* values lead to intervals that allow for life to have been formed more recently than 4.5 billion years ago, In short, the data analyzed by SG are consistent with the hypothesis that life may have formed after the Earth’s formation.

## Reviewers’ comments

### Reviewer’s report 1: Yuri Wolf, Institute of Cytology and Genetics, Novisibirsk, Russia

In a recent paper Sharov & Gordon (SG) infer the timeline of the growth of genomic complexity in the history of life and extrapolate the trend into the past. Strikingly, their analysis seems to contradict the origin of life on Earth since the x-intercept of the extrapolation indicates the age at least twice that of the Earth crust solidification. Here Marzban et al. offer criticism of this conclusion on the grounds of the SG failure to take into account the uncertainties in the estimates of the age of origin of major taxa.

The authors give a brief but fair tally of multiple problems with the SG analysis other than the focus of this work. I would like to emphasize that criticism on technical grounds by Marzban et al. does not imply that everything else is right about the SG approach. I consider this paper as an important and useful tutorial on regression using the problematic SG analysis as a teaching point, rather than an earnest attempt to show that the origin of Earth before life is compatible with the available data.

#### ***Two comments on the presentation***

- The authors use the term “measurement error models” (p. 6 of the manuscript). The related technique is also often referred to as “errors-in-variables models” and “Deming regression”: http://en.wikipedia.org/wiki/Errors-in-variables_modelhttp://en.wikipedia.org/wiki/Deming_regression (Wikipedia articles are cited here as evidence of popular usage, not as primary sources). It might be helpful to mention these terms along with the authors’ preferred name to facilitate recognition by the readers.

- The discussion of the relative likelihood of values within the prediction interval (p. 18 of the manuscript) is, in my opinion, either too superficial or superfluous. It would be interesting to see a full analysis of the relative likelihood of Age = 4.5 vs Age = 8.5 (preferably taking into account that errors in age estimates are not homoscedastic); otherwise it would be sufficient just to state that Age = 4.5 lies within the prediction interval and thus cannot be rejected.

#### ***A minor technical point***

- The authors call the relationship of inferred genome complexity with the estimated dates of the origin of lineage “logarithmic” (p. 5 of the manuscript). Actually, a linear relationship between the logarithm of genome size and the transition date indicates exponential relationship between the genome size and the date (since the date is, unquestionably, the argument here).

Authors’ reply 1: *We agree with the reviewer in that our analysis can be considered a tutorial on measurement error models. However, that does not reduce our conclusion that the data employed by SG do not support the hypothesis that life is older than Earth (even though it may be.)*

We also agree with the reviewer in that what we call “measurement error models” are known by a variety of different names in other fields. We may also add principal axis regression as an alternative method for taking errors-in-x into account.

Regarding the “superficial” discussion of prediction intervals, we believe it is important to include in the paper. In our own experience, that point is often missed, even among professional statisticians.

The reviewer is correct in that the relationship in question is exponential (and not logarithmic). The correction has been made.

### Reviewer’s report 2: J. Peter Gogarten, Biotechnology Services Center, University of Connecticut, Storrs, CT. USA

In their article Caren Marzban, Raju Viswanathan and Ulvi Yurtsever discuss problems that arise in extrapolation when errors and uncertainty in the data are not properly accounted for. They use work from Sharov [1] and Sharov and Gordon Sharov and Gordon [2] as an example. The work of Sharov and Gordon claims to find support for life originating a long time before Earth formed. Sharov and Gordon correlate the logarithm of estimates of the size of functional genomes to time of occurrence of the group. In his published review of the work (accompanying [1]) Chris Adami concludes “This paper is an example of how not to analyze data.” Indeed, there is much to criticize in the work by Sharov and Gordon examples are the reviews by Eugene Koonin, Chris Adami and Arcady Mushegian that accompanied the publication of [1]. The present article by Marzban et al. summarizes some of the criticism in a single paragraph and then focuses on the extrapolation to 1 bp complexity, or the x-axis intercept of the extrapolation. In their analysis Marzban et al. use the time and complexity estimates of Sharov and Gordon as starting point and then show that taking uncertainty of the time estimates into account leads to very large confidence and prediction intervals for the x-axis intercept. These intervals include 4.5 billion years BP, thus further invalidating Sharov and Gordon’s analysis. Marzban et al. give a good description of the problems associated with extrapolation from noisy data. In particular, they remind us that added noise leads to smaller slopes of the regression line. Their argument should be accessible to readers who are not statisticians. The only improvement I suggest concerns Figure 1. An example with more data points, and possibly with two levels of noise added (using a third color) might be more convincing to a casual reader, who might see a bias in in the noise added in Figure 1 (all *x*-values for low *y*-values happen to be increased due to the added noise).

Authors’ reply 2: *We have now revised the figure so that it does not display the bias pointed out by the reviewer. The revised figure also shows two levels of noise.*

### Reviewer’s report 3: Christoph Adami, Microbiology and Molecular Genetics, and Physics and Astronomy, Michigan State University. USA

This reviewer provided no comments for publication.

## Abbreviations

SG: Sharov [1], and/or Sharov and Gordon [2].

## Competing interests

The authors declare that they have no competing interests.

## Authors’ contributions

CM performed the statistical analysis, with significant feedback and discussion provided by RV and UY. All authors read and approved the final manuscript.
